# Google effects on memory: a meta-analytical review of the media effects of intensive Internet search behavior

**DOI:** 10.3389/fpubh.2024.1332030

**Published:** 2024-01-18

**Authors:** Chen Gong, Yang Yang

**Affiliations:** ^1^School of Journalism, Fudan University, Shanghai, China; ^2^College of Media and Arts, Shanghai University of Sport, Shanghai, China; ^3^College of Communication, Shanghai Lida University, Shanghai, China

**Keywords:** Google effects, meta-analysis, Internet use, cognition and memory, media effects

## Abstract

People are increasingly using the web for fact-checking and other forms of information seeking. The “Google effects” refers to the idea that individuals rely on the Internet as a source of knowledge rather than remembering it for themselves. However, few literature review have yet comprehensively examined the media effects of this intensive Internet search behavior. In this study, by carrying out meta-analysis, we found that google effects is closely associated with cognitive load, behavioral phenotype and cognitive self-esteem. And this phenomenon is also more likely to happen while using a mobile phone to browse the Internet rather than a computer. People with a larger knowledge base are less susceptible to the consequences of Internet use than those with a smaller knowledge base. The media effect was stronger for persons who had used the Internet before than for those who had not. And meta-analyses show that participants in North America (parameter = −1.0365, 95%CI = [−1.8758, −0.1972], *p* < 0.05) are more susceptible to frequent Internet search behavior relative to other regions. Overall, google effects on memory challenges the way individuals seek and read information, and it may lead to changes in cognitive and memory mechanisms.

## 1 Introduction

People are increasingly using the web for fact-checking and other forms of information seeking. The “Google effect” (1) refers to the idea that individuals rely on the Internet as a source of knowledge rather than remembering it for themselves. Humans may be less eager to answer inquiries willingly as a result of Internet access, preferring to utilize search engines instead.

Based on Ebbinghau (2) seminal work on the memory curve, it can be observed that a significant portion of the information that an individual reads is progressively forgotten, with the most rapid decline occurring on the initial day. Nowadays, the advent of the Internet era has significantly facilitated the process by which individuals obtain information, resulting in an increased ease of access. Simultaneously, the rate at which individuals forget information has experienced a notable acceleration. Based on findings in the field of neuroscience, it has been observed that the strength of memory is directly related to the frequency of memory retrieval. This implies that the hippocampus, as the primary processor of information, plays a crucial role in storing individuals’ memories, while the capacity to access these memories remains constant (3).

Nevertheless, the advent of search engines has rendered the capacity to retrieve certain pieces of information obsolete. After individuals have acquired proficiency in the cognitive process of use the search engine “Google” to retrieve information, they can discontinue the practice of performing the remaining content to memory, resulting in a gradual reduction in their reliance on external memory for stated material. The publication of an original research article by Sparrow et al. (1) in the esteemed journal Science has garnered significant interest, resulting in a substantial number of citations thus far. In this article, Sparrow explicitly asserted that individuals exhibit a readiness to contemplate desktops when confronted with difficult problems. And when people anticipate future access to information, they have lower information memory rates and a better recall of where to obtain the information. For instance, if they need to know the price of a commonly used everyday item, learn how to perform complex computer skills, or simply want to remember the name of the singer of a song they are listening to, they only need to turn on their computer, mobile phone, or any Internet-connected device with a search function, and they can find the answer instantly. It has become so ingrained in researchers to seek answers to any problem as soon as it arises that they may experience withdrawal if they are unable to find one immediately. And it is difficult to recall how people obtained information before the Internet became a pervasive part of our daily lives, as well as how information was discovered prior to the invention of the Internet. In this way, the Internet has a type of external or transactive memory in which data is stored externally.

Based on the transactive memory system, Kahn and Martinez (4) examined the relationship between the concepts of “Google’s effect on memory” and “cognitive self-esteem” in the interpersonal contexts of snapchat and text messaging as a complement to Sparrow et al. (1). The cognitive hypothesis regarding the effect of Internet use on memory is that people use computers as “transactive memory companions.” Wegner (5) was the first to propose an interactive memory system for encoding, storing, retrieving, and communicating information from numerous fields of knowledge. This division of labor typically occurs in close proximity (6). In additional ways, external memory can serve as a source or target for cognitive offloading (7). For this reason, many experts assert that the Internet’s influence on memory and associated studies are limited to “technology partners” (8–11).

In the fields of brain science and neuroimage science, researchers are accumulating evidence for “Google effect.” Most directly, the cognitive effects of Internet use may result in changes to brain structure, such as the density of gray matter (12). Due to the lack of scientific literature examining the neural correlations involved in online information processing, his experiments have contributed to the advancement of brain science research on the phenomenon of memory loss caused by Internet use. In addition to long-term Internet search behavior, short-term behavior has the potential to increase Internet reliance (13). In Wang et al. (13) experiment, a comparison of post-test and pre-test data on changes in brain activation as well as a comparison of self-reported changes in neural impulses to unknown trials were obtained by setting up participants for Internet search training, further demonstrating that after training on Internet search questions (even for a short period of time), participants had a greater impulse to use the search engine again when presented with a query.

Traditional social science disciplines have considered “Google effect.” Several social science researchers, in conjunction with BBC television, predicted that search engines such as Google would cause a fundamental shift in the way people search and comprehend information in the twenty-first century (14, 15), which prompted sociologists and anthropologists to study this phenomenon. Parslow (16) also discovered that when the Internet was present in the Head Start environment, it was more difficult for children to concentrate and engage with lengthy texts. However, Parslow (16) later statement that “the Internet and related technologies are actually good for the brain” lacks evidence to support it, at least not from his short article, even though there is similar evidence that (1) Only on other social groupings has the Internet reduced reliance (17); and (2) Having effective integrated skills is more valuable than biological memory (18). Moreover, it is not only the Internet that can prevent people from becoming dependent (19).

Different academics have analyzed “Google effect” on various age groups to determine whether or not it is universal across all ages. Danovitch (20) summarized youths’ understanding of the use of web devices from birth to age eight, revealing that children’s judgments about the Internet are influenced by their own personal experiences (e.g., the belief that they can only play games on the Internet) and that children are less likely to rely on the Internet for answers because they take longer to trust the information they obtain from the Internet. However, as they become adults, the younger age group is more likely to anticipate memory changes (21) because they perceive themselves as more adept Internet users, whereas the more experienced are more likely to experience a decline in their internal memory. Even if older people have excellent Internet search skills, “Google effect” is unlikely to be present, especially for those with cognitive impairments (22). Slegers et al. (22) extended the study to a group of cognitively normal older individuals with no Internet experience and found no evidence that learning Internet search skills has any cognitive effect on inexperienced older individuals. However, older people can also take advantage of the Internet’s emerging opportunities to receive new sources of cognitive stimulation, even more so than younger people (23), suggesting that “Google Effect” has different effects on older and younger individuals. Notably, there have also been ethnically specific studies, such as the study of the attention span of Finns when using the Internet (21).

In summary, the inconsistencies in previous research constitute a formidable barrier to understanding gender differences in media effects of frequent Internet search behavior. The key question addressed in this study is what factors may moderate, amplify, attenuate, or conceal the potential differences in “Google effects.” In prior findings (24), meta-analysis is an ideal technique for synthesizing group differences and identifying potential contributors to inconsistency or heterogeneity. Consequently, the present study adopted a meta-analytic approach and sought to synthesize the magnitude and direction of potential differences in effects of frequent Internet search behavior, as well as to identify potential moderators of those differences.

### 1.1 Potential factors related to inconsistent findings

#### 1.1.1 Region

The presence of diverse cultures and work habits across various regions can potentially influence the manner in which individuals with varying cognitive frameworks utilize search engines for information retrieval. Consequently, this can have implications for the assessment of search engines’ influence on individuals’ perceptions (25). Therefore, we explore whether region (e.g., North America, Europe, or Asia) moderates the relationship between frequent Internet search behavior and cognitive effects.

#### 1.1.2 Gender

The literature reviewed in this paper does not currently include any research that has specifically investigated the Google effects in relation to gender differences. Nevertheless, considering that the majority of the articles under examination provide data on the gender distribution of individuals, we have properly considered this as a plausible variable that could have impacted the outcomes. Due to significant variations in the gender distribution among the studies (26, 27), we incorporated a column indicating the percentage of males in the subsequent meta-analysis figures, in order to mitigate potential discrepancies across publications.

#### 1.1.3 Type of sample

Different sample features may be related to conflicting findings on gender differences in google effects on memory. For instance, with an adolescent sample, Yu et al. (28) found a large gender difference in search behavior leading to cognitive change. With a community sample of adults, however, Hamilton et al. (25) and Kahn and Martinez (4) did not detect a significant gender difference. Moreover, Kamin et al. (27) and Yu et al. (28) showed that gender differences in search behavior leading to cognitive change were smaller in online databases samples than in college samples. Sample characteristics (e.g., college vs. public databases sample) speak to the strictness of a participant’s immediate situation—such as the behavioral restriction within the living community—which may affect the measurement of results. Therefore, it is imperative to investigate whether the nature of the sample or cohort might influence the association between gender and google effects on memory.

#### 1.1.4 Experiment measurement type

The researchers conducting this meta-analysis have thoroughly examined and categorized the various types of experiments discussed in the articles pertaining to the Google effects on memory. These experiments have been classified into five distinct categories, denoted as cognitive load (cognitive load theory, CLT, is an instructional framework grounded in our understanding of human cognition. It relies on a cognitive architecture comprising a restricted working memory, which is partially isolated from processing units responsible for visual and auditory information. This working memory engages in interactions with an unlimited long-term memory) (1, 29, 30), cognitive measurement (the development of new automated data collection methods, the application of cognitive psychology concepts and methods to reduce survey measurement error) (17, 25, 31), behavioral phenotype (they are recognizable patterns of behavior-syndromes) (1, 32, 33), cognitive self-esteem (individuals with greater confidence in their own intelligence, memory and ability would hold the belief that information is easily accessible, such as through the use of a search engine) (4, 8) and psychophysiology (measurements with physiological instruments, e.g., EEG, fMRI, etc.) (13, 17). The subsequent sections of the meta-analysis further categorizes these five groups to derive more accurate and specific conclusions.

#### 1.1.5 Age

Previous studies have demonstrated that the extent and orientation of differences in search behavior leading to cognitive change may vary throughout the stages of adolescence, young adulthood, and middle age. For instance, Yu et al. (28) conducted a study examining problematic Internet use among a sample of adolescents (*M* = 15.33, *SD* = 0.47). Similarly, Sanchiz et al. (34) conducted a study investigating the impact of frequent search engine usage on human memory cognition. They employed a cognitive aging-related experimental design, which included both an experimental group (*M* = 66.00 years old, *SD* = 3.45) and a control group (*M* = 21.28 years old, *SD* = 1.78). There exists a substantial disparity in the effect sizes observed in the outcomes of the two previously mentioned experiments. Therefore, age may also influence the media effects brought by Internet search behavior.

## 2 Methods

### 2.1 Selection of studies

A literature search was conducted for studies that had examined google effects on memory published up to June 2023. A computer-based search was conducted using ACM Digital library, PsycINFO, Web of Science, IEEE Xplore, and Scopus. To mitigate the possibility of unintentionally excluding literature, the entire search procedure was replicated three times at one-month intervals in these databases. Records containing the search string in their titles or abstracts were located and the search string can be found in https://osf.io/b8d94/ (see Supplementary material S1 as well). For the search string, it was divided by the author into three distinct sections: the object or phenomenon, the method or tool employed, and the journal type. The most frequently searched keywords were entered into each section, employing logical linkers such as “AND” and “OR” and wildcards like “*” to facilitate the search for multiple forms of the term throughout the screening procedure. For instance, the input “memor*” may return results for terms such as “memory,” “memorize,” and so on, but not words like “distributed memory.” To link each input, an “OR” link was necessary due to their relatively similar meanings. The initial search identified 908 records and 35 studies in 22 different articles were finally considered. The meta-analysis incorporated studies that satisfied the following set of criteria: (1) The decision to use English as the language of publication for this study was based on previous research findings that indicated a lack of substantial evidence suggesting any systematic bias resulting from the restriction to English language (35) in meta-analyses; (2) was published in peer-reviewed journals or conferences; (3) provided comparisons between various experiments in terms of google effects on memory, and also reported or made it possible to calculate the effect size (Cohen’s *d*) for these comparisons; (4) Experiments must be well designed methodologically. We refer to the criteria related to assessing experiments quality in Chen et al. (36), for example, “was the study population clearly specified and defined” and “well developed with reporting validity and/or reliability.” And experiments with qualitative or quantitative analysis of evidence to support conclusions, and if part of the experiment involves a moral and ethical test, only ethically certified experiments will be considered.

We did not include unpublished findings for the following reasons: (1) Only original research and quantitative literature review could be selected, and it should not be a novel, communication letters or editorial report, etc.; (2) Studies that do not focus directly on the methods used for memory or cognition change on Internet use, but merely refer to the various effects of the Internet; (3) Studies not discussing the effect of using search engines but only focusing on the different methods to use them; (4) Reports on the same subject that have been published more than once (37). So in this research, the most comprehensive iteration of the study was included when multiple reports of the study were found in various journals; (5) other spontaneous surveys in the literature (e.g., no defined research objectives; no defined search process; no defined data collection process).

### 2.2 Coding of studies

The discrepancies among the raters were resolved through engaging in discussions with the first and second authors. The following information was extracted from each of the included studies: paper ID and study ID; authors; publication year; regions (north America, Europe, Asia); age; male, female and percentage of males; treatment and control group sample size; experimental measurement indicators; research type (cognitive load, cognitive measurement, behavioral phenotype, cognitive self-esteem, psychophysiology); and Cohen’s *d* or the information for computing Cohen’s *d*. The two coders were trained to code a set of articles that had been expertcoded for another study prior to beginning formal coding. Once the coders had acquired experience in coding articles for meta-analyses, they commenced the formal coding process. The reliability of the final intercoder varied between 0.91 and 1.00 (Krippendorff’s alpha). Discrepancies were resolved through discussions. For included studies that did not directly report Cohen’s *d*, means and standard deviations of experiment and control groups for were extracted and used to calculate d. Where necessary, we changed the sign of the published Cohen’s *d* to ensure that all effect sizes were consistent.

### 2.3 Data analyses

The data analyses were performed using R (38) and the metafor package (39). We conducted mean effect size analyses, heterogeneity tests and other required analysis. Initially, to calculate a comprehensive mean effect size, we employed a random-effects model as we anticipated substantial variability in effect sizes across studies. And the determination of the effect size of Cohen’s *d* was derived from (40) guidelines, which established 0.20 as the threshold for a small effect, 0.50 for a medium effect, and 0.80 for a large effect.

Furthermore, the evaluation of unexplained heterogeneity is an essential component in the process of conducting a meta-analysis. Both random sampling error, which refers to the variability observed within a study, and systematic study features, such as the characteristics of the samples, the selection of measurement instruments, and the type of publication, have the potential to contribute to the heterogeneity observed among studies. The outcomes of the heterogeneity test have a direct impact on the choice of statistical models, specifically fixed-effects versus random-effects, when conducting an analysis of effect sizes. The Cochrane Q statistic and the I2 index are frequently employed in the evaluation of the statistical significance of unexplained heterogeneity (41). Based on the I2 statistic, mild heterogeneity is indicated by a range of 0–40%, moderate heterogeneity is within the range of 40–60%, greater heterogeneity is represented by a range of 50–90%, and great heterogeneity is denoted by a range of 75–100%.

Thirdly, we conducted moderator analyses to investigate the potential factors that may have contributed to the variability observed in previous research findings. Several potential moderators were examined (e.g., publication year, region, age etc.). We suspected that if there is a statistically significant moderating effect was found for a categorical moderator comprising more than two levels, a post-hoc comparisons to examine the gaps between the levels within the moderator would be processed. To reduce the risk of Type one errors, we employed Bonferroni corrections during the post-hoc pairwise comparisons.

Publication bias for articles is generally illustrated by a funnel plot (42), which estimated that if there is a certain relative symmetry between the studies on both sides of the vertical line of the combined effect size, and with a non-significant *p*-value (i.e., greater than 0.050) indicating insufficient evidence for publication bias (43).

## 3 Results

### 3.1 Study selection

**Figure 1:**
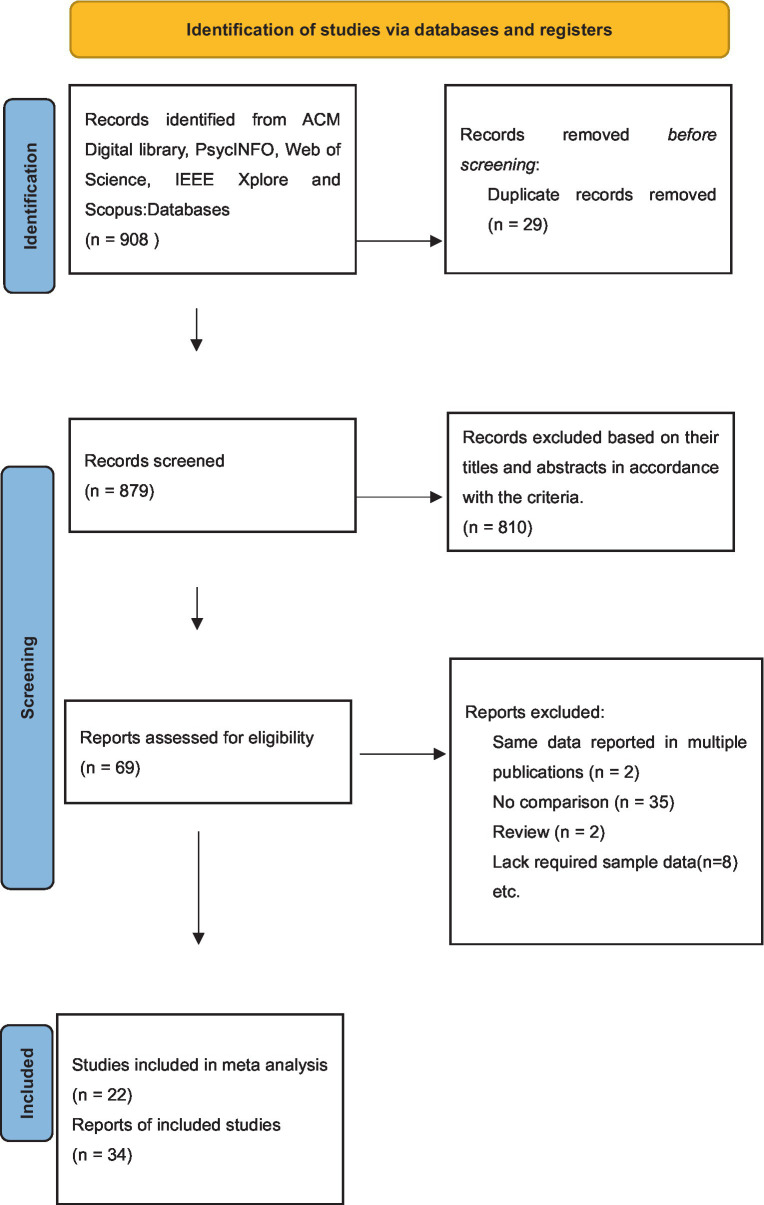
PRISMA study selection flowchart.

The selection of studies for this meta-analysis is illustrated graphically in Figure 1. The initial search identified 908 records from the five (ACM Digital library, PsycINFO, Web of Science, IEEE Xplore and Scopus) databases. After removing duplicates, 879 studies remained. After excluding studies based on their titles and abstracts in accordance with the criteria described in the method section, 69 studies were included in the full-text review. Twenty two out of 69 full texts were determined to meet the meta-analysis selection criteria. In addition, the reference lists of all the chosen articles were examined for additional relevant studies, but no additional records were found. Among these 22 articles, seven contained multiple independent sub-studies with samples (multiple tasks in the same sample or overlap of participant sample) (1, 17, 25, 31, 44, 45), resulting in 35 independent comparisons of google effects on memory. More details of initial records and considered articles can be found in Supplementary materials S3, S4.

### 3.2 Descriptive characteristics of the studies included

The 22 selected articles involved a total of 30,889 participants with their ages ranging from 12 to 89 years old. The dates of publications ranged from 2011 to 2021. The reported effect size (Cohen’s *d*) of cognitive effects by frequent Internet search behavior ranged from −0.85 (31) to 4.38 (46), with a positive effect size indicating higher levels of google effects on memory. The descriptive characteristics of the studies are shown in https://osf.io/b8d94/.

### 3.3 Overall analysis

Using a random-effects model, the average effect size and its 95% confidence interval were estimated. The pooled d indicated a moderate but statistically significant effect size. The forest plot is described in appendix in our open data platform.

### 3.4 Subgroup analysis

**Figure 2:**
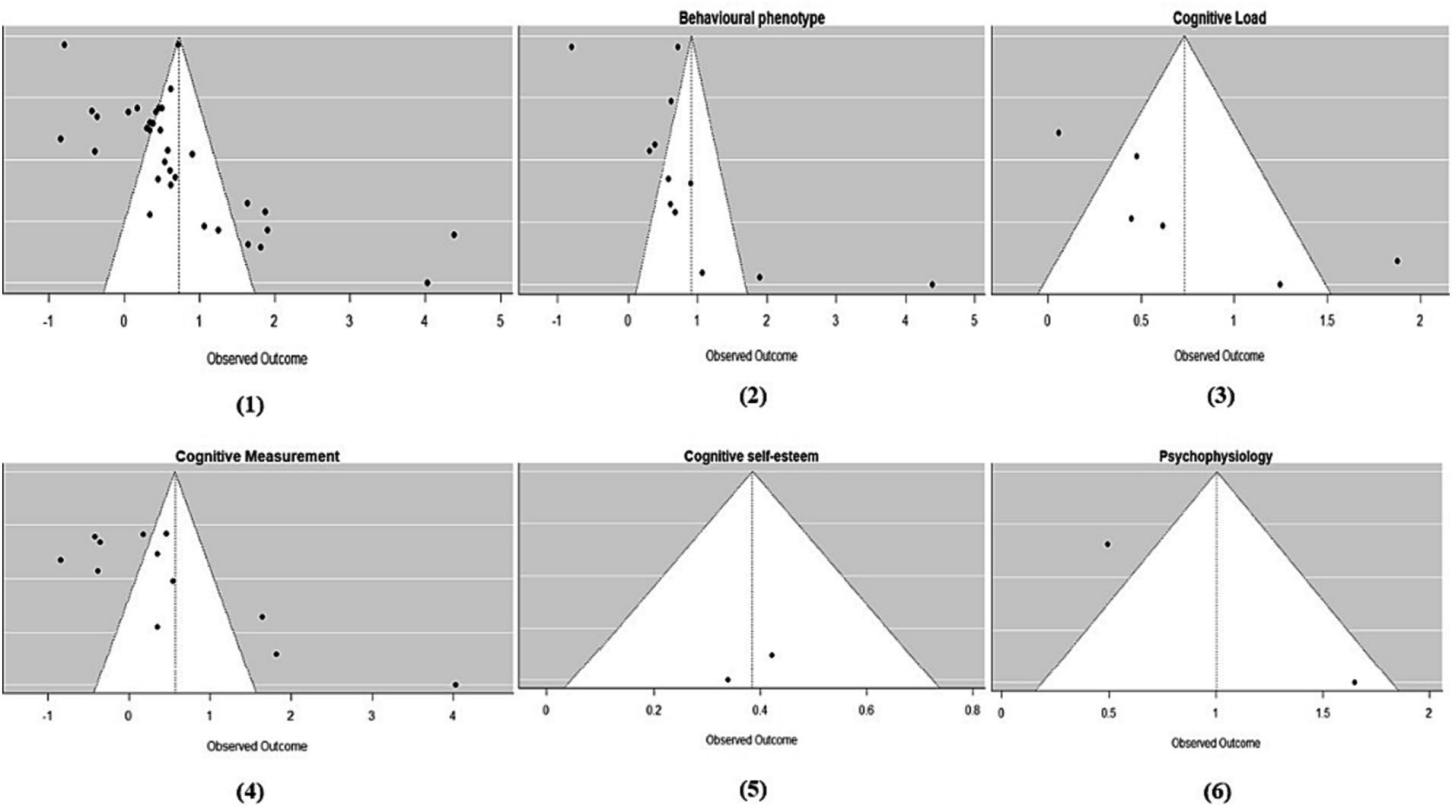
Funnel plot for checking publication bias. (1) Is the funnel plot for overall publication bias, (2)~(6) are plots for subgroups publication bias.

**Figure 3:**
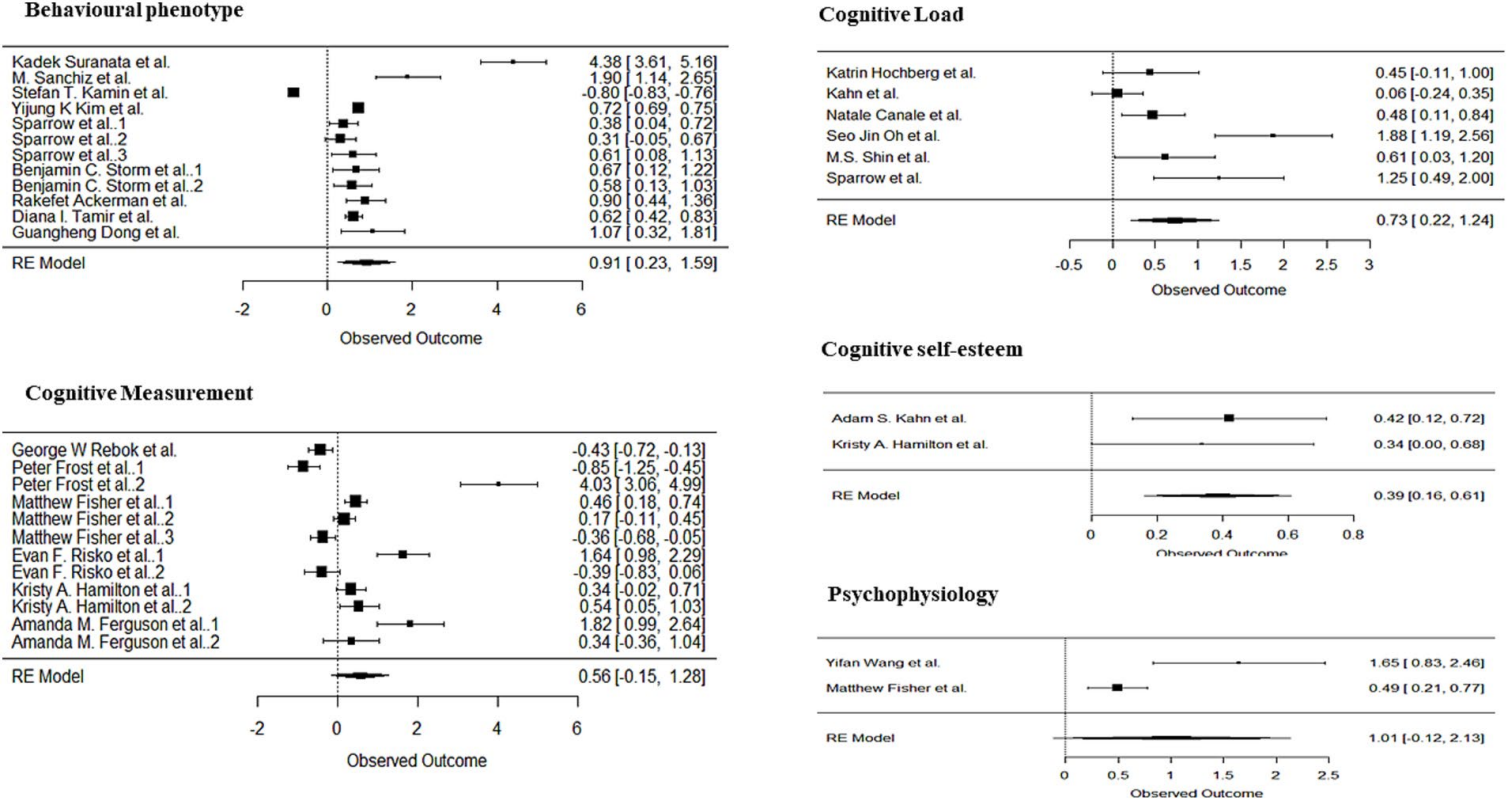
Subgroup analysis.

Since the experiments included in the meta-analysis were mainly classified into five categories of research type: cognitive load, cognitive measurement, behavioral phenotype, cognitive self-esteem and psychophysiology. we did subgroup analyses of the included literature and the results are shown in Figure 2. For the cognitive load subgroup, observed outcome = 0.73, 95%CI = [0.22, 1.24], which shows the estimates of cognitive load are positively influenced, indicating that this factor has a significant positive effect on the variables analyzed. And this is similar to behavioral phenotype subgroup and cognitive self-esteem subgroup, with observed outcome = 0.39, 95%CI = [0.16, 0.61] and observed outcome = 0.91, 95%CI = [0.23, 1.59] respectively. For the cognitive measurement subgroup, observed outcome = 0.56, 95%CI = [−0.15, 1.28], although the estimates for the cognitive measures were positive, 95%CI included zero, so it was not possible to conclude whether this factor had a significant effect on the variables analyzed. And this is similar to psychophysiology subgroup, with observed outcome = 1.01, 95%CI = [−0.12, 2.13] (for more details, see Figure 3).

### 3.5 Moderator analyses

The results of moderator analyses are shown in Table 1. Overall, results indicated that only one variable contributed significantly to the heterogeneity of effect sizes: region (*Q* = 6.1997, *p* < 0.050) accounting for 13.18% of the total heterogeneity. We argued that there are other omitted variables that are not included in the regression and are therefore absorbed into the intercept term, resulting in the intercept being significant but some variables not being significant. Specifically, for region, the pooled d from Europe (*d* = −1.1063, 95%CI = [−2.3157, 0.103]) was larger (take an absolute value) than that from North America (*d* = −1.0365, 95%CI = [−1.8758, −0.1972], *p* < 0.05). One possible hypothesis posits that there exists a positive correlation between the frequency of Internet search engine utilization and both the availability of broadband connections in the area and the local GDP (47). Furthermore, this discovery is consistent with the results of a recent study (48) that examined populations across 21 regions and countries and discovered that the impact of social media fatigue and Internet addiction was more pronounced in Europe compared to the America and Asia. For research type, the pooled d from cognitive self-esteem (*d* = −0.5262, 95%CI = [−2.1533, 1.1009]) was the greatest (take an absolute value) and the parameter from psychophysiology (*d* = 0.1297, 95%CI = [−1.5356, 1.7950]) was the smallest (see Table 1 for more details).

**Table 1:** Moderator analyses.

	Parameter	SE	*z*	95%CI		*Q*	df	*R*^2^
				Lower limit	Upper limit			
Pub.year	0.0172	0.0632	0.272	−0.1067	0.1411	0.074	1	0.00%
Region						6.1997	2	13.18%
EU	−1.1063	0.617	−1.793	−2.3157	0.103			
NA	−1.0365*	0.4282	−2.42	−1.8758	−0.1972			
Percentage of males	−0.0167	1.842	−0.009	−3.6269	3.5936	0.0001	1	0.00%
Age								
Sample size	−0.0138	0.0122	−1.137	−0.0377	0.01	1.293	1	0.83%
Type	−0.0000	0.0001	−0.677	−0.0001	0.0001	0.4579	1	0.00%
CL							4	0.00%
CM	−0.1382	0.5534	−0.25	−1.2228	0.9465			
CSE	−0.3563	0.5534	−0.791	−1.2396	0.5269			
Psy	−0.5262	0.8302	−0.634	−2.1533	1.1009			
	0.1297	0.8497	0.1527	−1.5356	1.7950			

*p < 0.05, **p < 0.01, ***p < 0.001. EU, Europe; NA, North America; CL, cognitive load; CM, cognitive measurement; CSE, cognitive self-esteem; Psy, psychophysiology; Pub.year, publication year.

### 3.6 Publication bias and sensitivity analysis

The analysis of publication bias for these articles is typically illustrated by a funnel plot, as shown in Figure 2. This funnel plot demonstrates that there is a certain degree of relative symmetry between the studies on both sides of the vertical line of the combined effect size, leading to the conclusion that publication bias is insignificant.

## 4 Discussion

Using meta-analytic techniques, this study provided a quantitative summary of the literature on media effects brought by frequent Internet search behavior prior to Jun 2023. Based on a random-effects model, our findings indicated that frequent Internet search behavior may lead to the change of cognitive load, behavioral phenotype and cognitive self-esteem. It was also discovered that one significant moderator (region) contributed to the heterogeneity of previous findings.

This paper will address two primary issues: How do people use their human memory when working on Internet-based projects? (2) How does the use of the Internet affect human cognition (this could be memory or a related concept)? These two questions are now answerable. To answer the first question, it is possible for people to forget where their thoughts end up and what is stored internally versus what is stored online when they use the internet to access information. Because the Internet is an example of a repository for interactive memory, people who operate on the Internet can easily rely on this memory. Since the advent of the Internet, people’s cognitive memories have changed. Even if the cognitive mechanisms that comprise the interactive memory system do not change, Internet users may begin to model characteristics of the Internet into their own self-perceptions, and they may believe that they are particularly adept at thinking and remembering information, despite the fact that the Internet is increasingly responsible for “remembering” information (1, 49). Moreover, when people search for information on the Internet while working on the Internet, they are more likely to use the Internet rather than their brain the next time they encounter that issue, and they retain pertinent information in an interesting way: they remember the Internet address where the pertinent information is stored (e.g., domain name, database, etc.). People who have searched the Internet for a solution to a problem, for example, will remember the website where they found the solution more vividly when they encounter the problem again (50), even if they have forgotten the precise essence of the problem for which they were searching.

To answer the second question, the effect of working on the Internet is an essential-to-phenomenal process that first may have caused some changes in brain structure or function, e.g., the act of using the Internet creates new connections in the brain’s synapses, causing further structural changes in the brain’s gray matter layer, and then these changes in brain plasticity cause individuals to misinterpret the content of transactive memories. People are more likely to return to the Internet and repeat the search process when confronted with a similar issue if they remember the location where the information was saved. Moreover, people’s perceptions of findability roughly predict the amount of time it will take them to find the information they need on the Internet, which increases their reliance on it laterally (38). Moreover, when searching for solutions on the Internet, rapid responses (answers that are obtained more quickly) are more likely to be convincing. People feel more confident when working in an Internet-accessible environment, and “Google effect” is stronger for those who have previously used the Internet. Race and age influence the “Google effect” differently.

Academic perspectives on this phenomenon range from support to concern, but the vast majority have considered ways to coexist with it. Basic research on the Google effect has been completed, and for future research directions, the primary focus will be on close integration with the social sciences (experimental psychology, situational memory, cognitive psychology, etc.); expanding the range of experimental subjects (sample size, expanding the age range of the population, e.g., the experimental phenomenon of memory effects caused by Internet use in the older adult is still unclear), and establishing universal e-learning environments. Determine the extent to which memory is affected by different Internet devices and the actual Internet environment (i.e., the presence of misleading information on the Internet) on human memory.

Over thousands of years, humans have demonstrated a remarkable capacity to employ new technology to grow and enhance their cognitive abilities; therefore, fear of new technologies is not new. Academics believe that the Internet’s benefits will ultimately outweigh any disadvantages it may have, even if the current era is unique and the Internet’s features are vastly different from those of previous technologies. In addition, by examining the potential costs and limitations of the Internet, individuals are in a better position to develop and modify the technology so that it is potentially more productive, less disruptive, and more consistent with the everyday goals and functions of human cognition.

There are some limitations on our present study. Due to the lack of solicitation for ongoing or unpublished studies from google effect researchers, there is a potential for the missing of certain relevant research that were not included in the databases searched. Second, while the current study identified significant moderators, it is possible that some moderators may have been neglected because they may not be highlighted during our coding process. Third, in moderators’ analysis, we found that the pooled d from Europe was larger than that from North America, we considered the region analysis could be optimized further (e.g., by including country-specific data). Fourth, funnel plots were employed to investigate publication bias in our current study. However, potential sources of bias may still exist. Subsequent related research could employ additional tests [e.g., Trim-and-Fill test (51, 52)] for further study.

Despite these limitations, our findings have various theoretical and practical implications. Regarding the former, this is possibly the first meta-analytical review on google effects, and it makes a significant academic contribution to the evidence base. This study is also carried out during the time that people may have more media and search engines exposure (53). Regarding practical implications, our findings may be useful for local government and teachers. The regional differences in susceptibility to frequent Internet search behavior may prompt policymakers to think about adapting some measures to reduce google effects on memory by influencing the way local people think if possible. One plausible strategy may involve a record of application usage durations and the distribution of individualized security alerts to particular user groups. Our research holds significant implications for a broad classroom teachers’ group, given the prevalence of mobile device usage (e.g., tablets) in the classroom today. While such devices are used, their extent of student usage must be strictly regulated. As illustrated by Hochberg et al. (26), we cannot simply infer if a higher learning efficacy can be achieved by using video analysis with tablets in comparison with traditional physics classes. So we also highly recommend the implementation of campaigns to raise public awareness of intensive Internet use behavior and promote media literacy. To mitigate the negative effects of this, future research may investigate some applicable psychological therapies such as mindfulness and positive thinking therapy. Additionally, correlations between specific search engines’ characteristics and individual differences, such as personality traits, could be investigated further to determine which population is most susceptible to google effects.

In sum, this is the first meta-analytical research review for google effects on memory. Frequent Internet search behavior was found to be closely associated with cognitive load, behavioral phenotype and cognitive self-esteem. And this phenomenon is also more likely to happen while using a mobile phone to browse the Internet rather than a computer. People with a larger knowledge base are less susceptible to the consequences of internet use than those with a smaller knowledge base. The media effect was stronger for persons who had used the Internet before than for those who had not. And meta-analyses show that participants in North America (parameter = −1.0365, 95%CI = [−1.8758, −0.1972], *p* < 0.05) are more susceptible to frequent Internet search behavior relative to other regions. Overall, google effects on memory challenges the way individuals seek and read information, and it may lead to changes in cognitive and memory mechanisms. A future update of the current meta-analysis can then be conducted when more research collecting and reporting year, type, method, gender, region, and subgroups differences becomes available.

## Data availability statement

The datasets presented in this study can be found in online repositories. The names of the repository/repositories and accession number(s) can be found in the article/Supplementary material.

## Author contributions

CG: Conceptualization, Data curation, Formal analysis, Methodology, Writing – original draft, Writing – review & editing. YY: Validation, Writing – original draft, Writing – review & editing.
