# Supplementary material for: Google effects on memory: a meta-analytical review of the media effects of intensive Internet search behavior
Source: Front Public Health. 2024 Jan 18;12:1332030. doi: 10.3389/fpubh.2024.1332030 (PMC10830778; doi:10.3389/fpubh.2024.1332030)

Supplementary material S1

Databases: ACM Digital library, PsycINFO, Web of Science, IEEE Xplore and Scopus

Search String:

**Inclusion/exclusion search string**

| Search Location | Objects/phenomenon | Tools/approach | Types/purposes of articles |
| --- | --- | --- | --- |
| TI(title) | memor* OR distributed memory OR distributed cognition OR offloaded cognition OR offloaded memory OR mind* OR cogniti* OR attention* | web* OR Internet* OR smartphone* OR google effect | compar* OR effect* OR impact* OR implicat* OR review* OR research* OR discuss* OR survey* |
| (((((( TI = (((memor* OR distributed memory OR distributed cognition OR offloaded cognition OR offloaded memory OR mind* OR cogniti* OR attention*)AND(web* OR Internet* OR smartphone* OR google effect))AND(compar* OR effect* OR impact* OR implicat* OR review* OR research* OR discuss* OR survey*)))))))) | | | |

In terms of the specific content of the search terms, as this article is about the effect of Google on human cognition and memory, the authors set the words in the “objects”

column to “memory”, “cognition,” “attention”, and their related derivative phrases, without setting the keywords for subjects such as the primary argument for avoiding using keywords like "human" is that other live creatures are unlikely to utilise the Internet for a variety of search activities. The words “web”, “Internet”, “phone”, and their derivative phrases were put in the “tools” column, and the particular phrase "Google effect" was added to search directly for scholarly articles that were almost suitable for this study. Finally, to provide a systematic and comprehensive overview of the type of literature to be searched, the authors keyed in usual keywords for the purpose or methodology of scientific papers, such as articles exploring impact, review articles, survey articles, original research articles, comparative experimental articles, and so on.

Subgroup Analysis-Influencing factors:


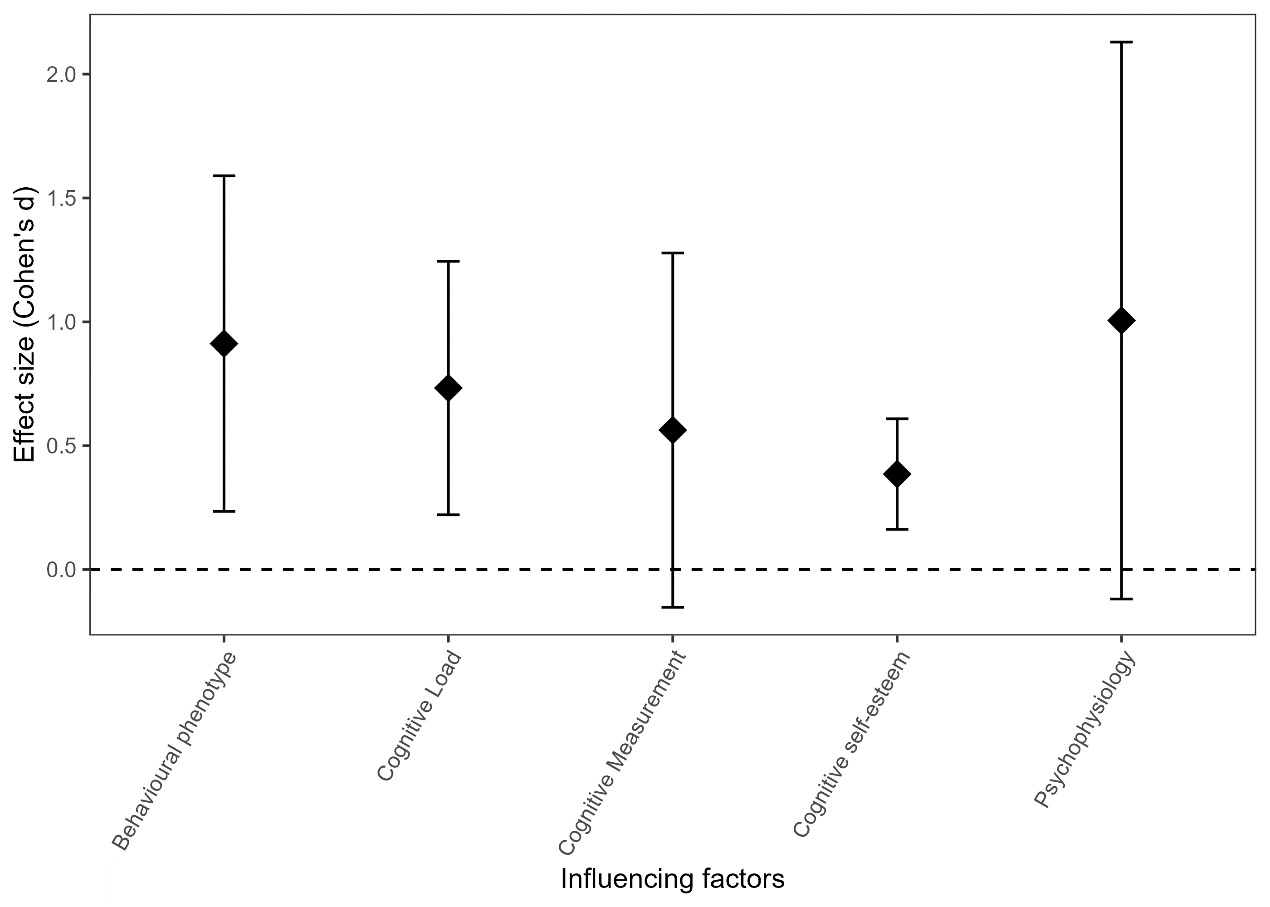


Subgroup Analysis:


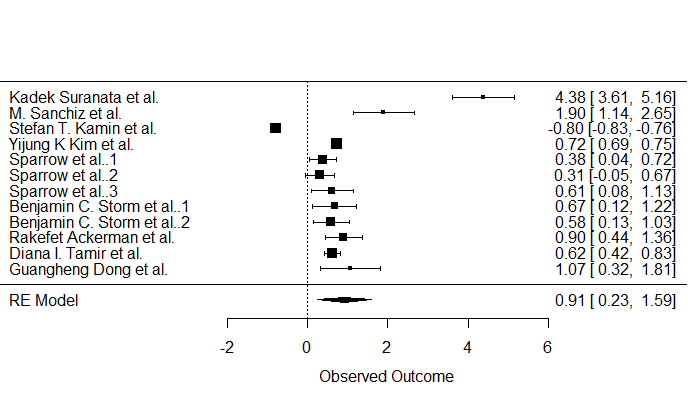


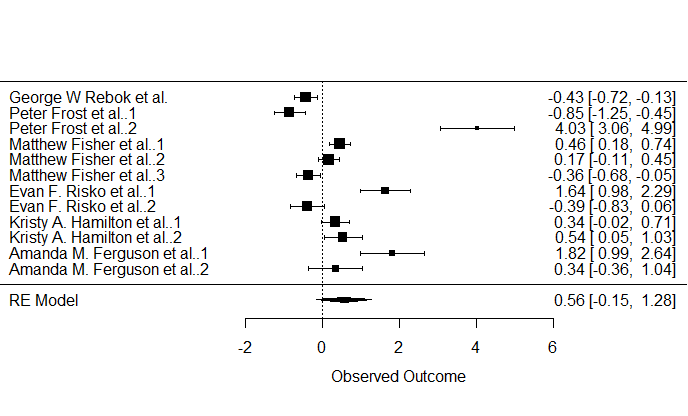


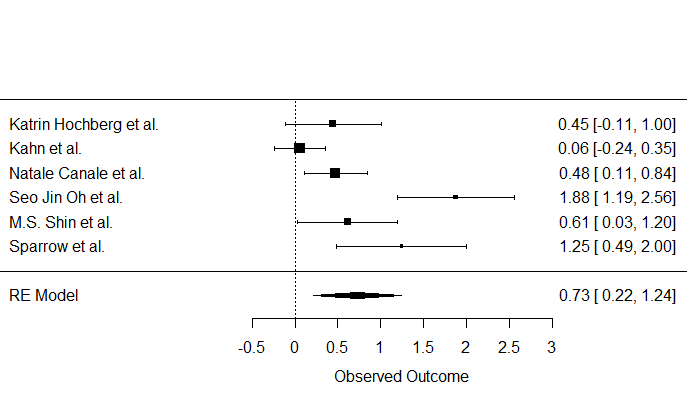


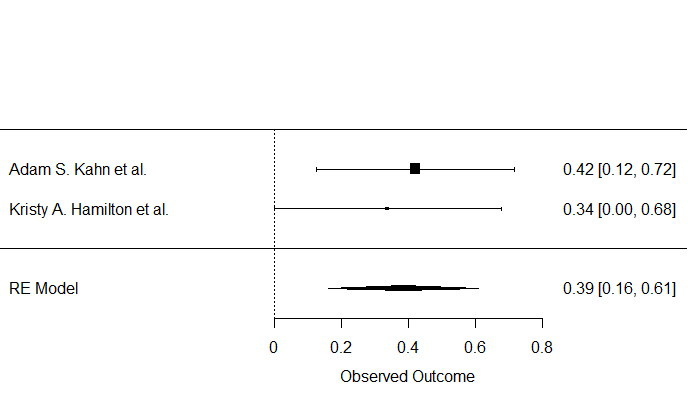


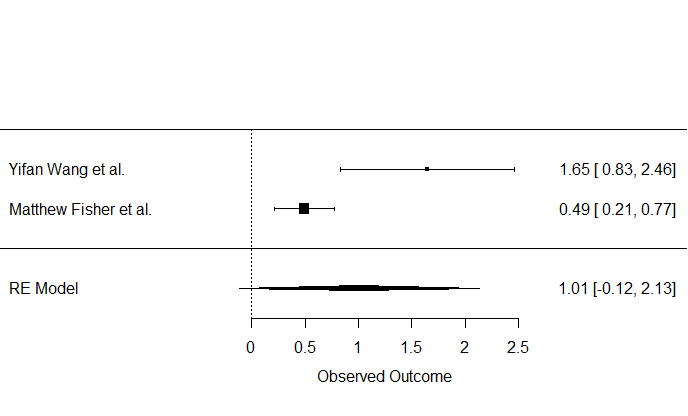


Overall Funnel plot：


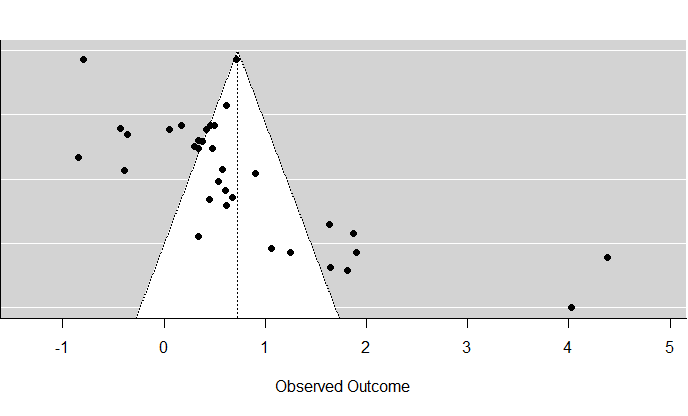


Subgroup Funnel plot:


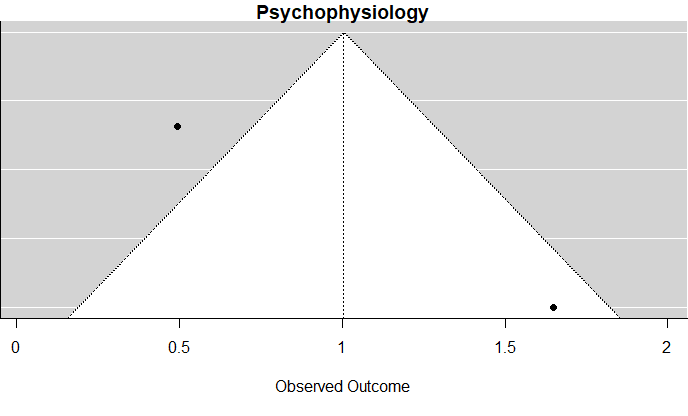

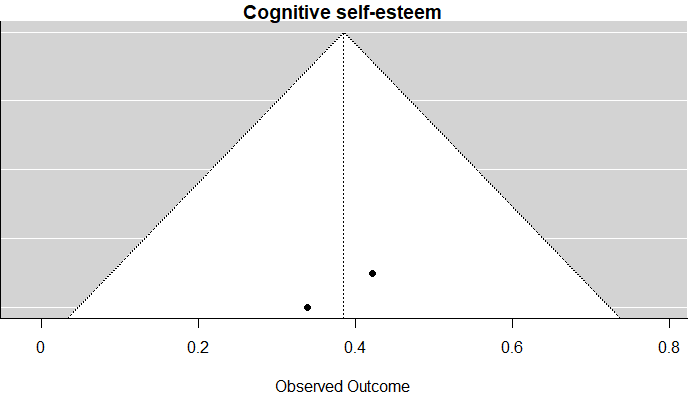

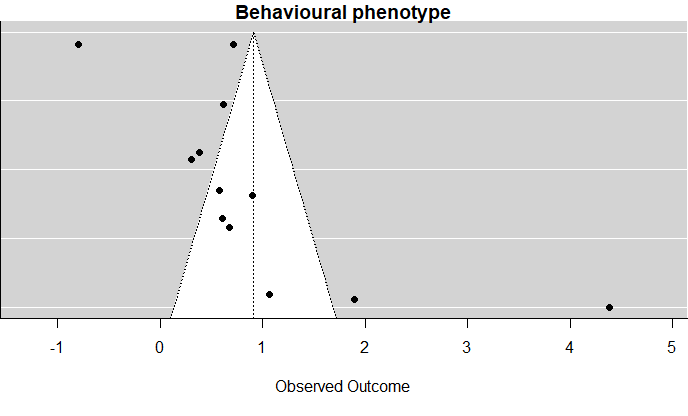

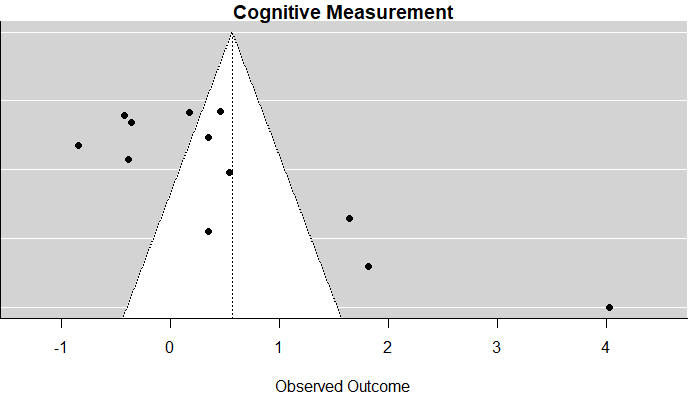


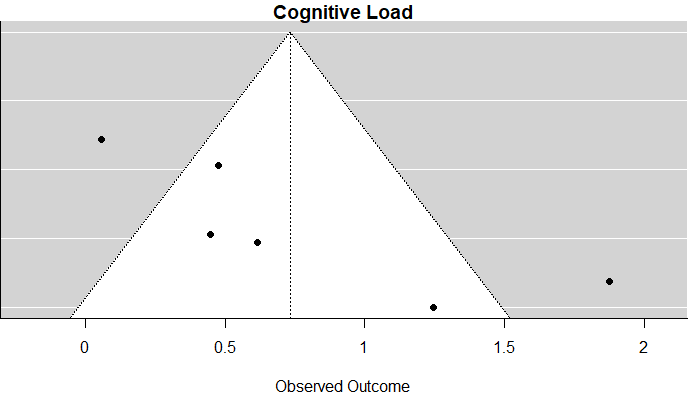


Potential influence analysis:


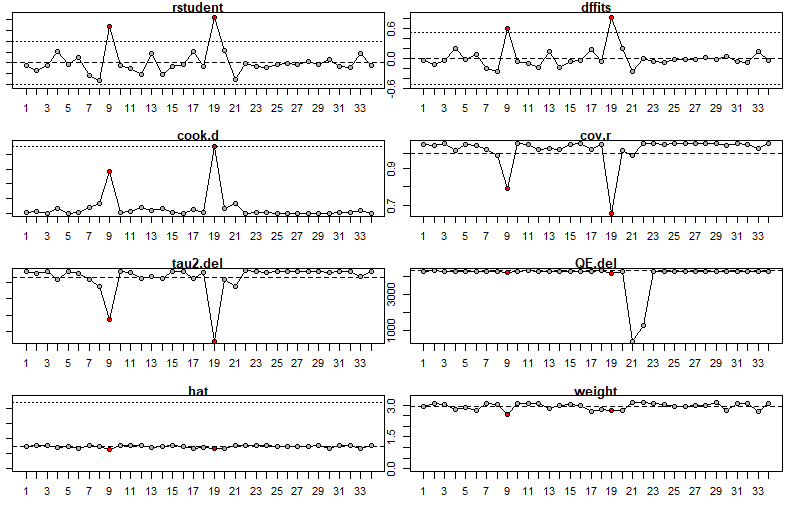

Supplement: Supplementary file 1 [file Data_Sheet_1.DOCX]
