# Supplementary material for: Google effects on memory: a meta-analytical review of the media effects of intensive Internet search behavior
Source: Front Public Health. 2024 Jan 18;12:1332030. doi: 10.3389/fpubh.2024.1332030 (PMC10830778; doi:10.3389/fpubh.2024.1332030)

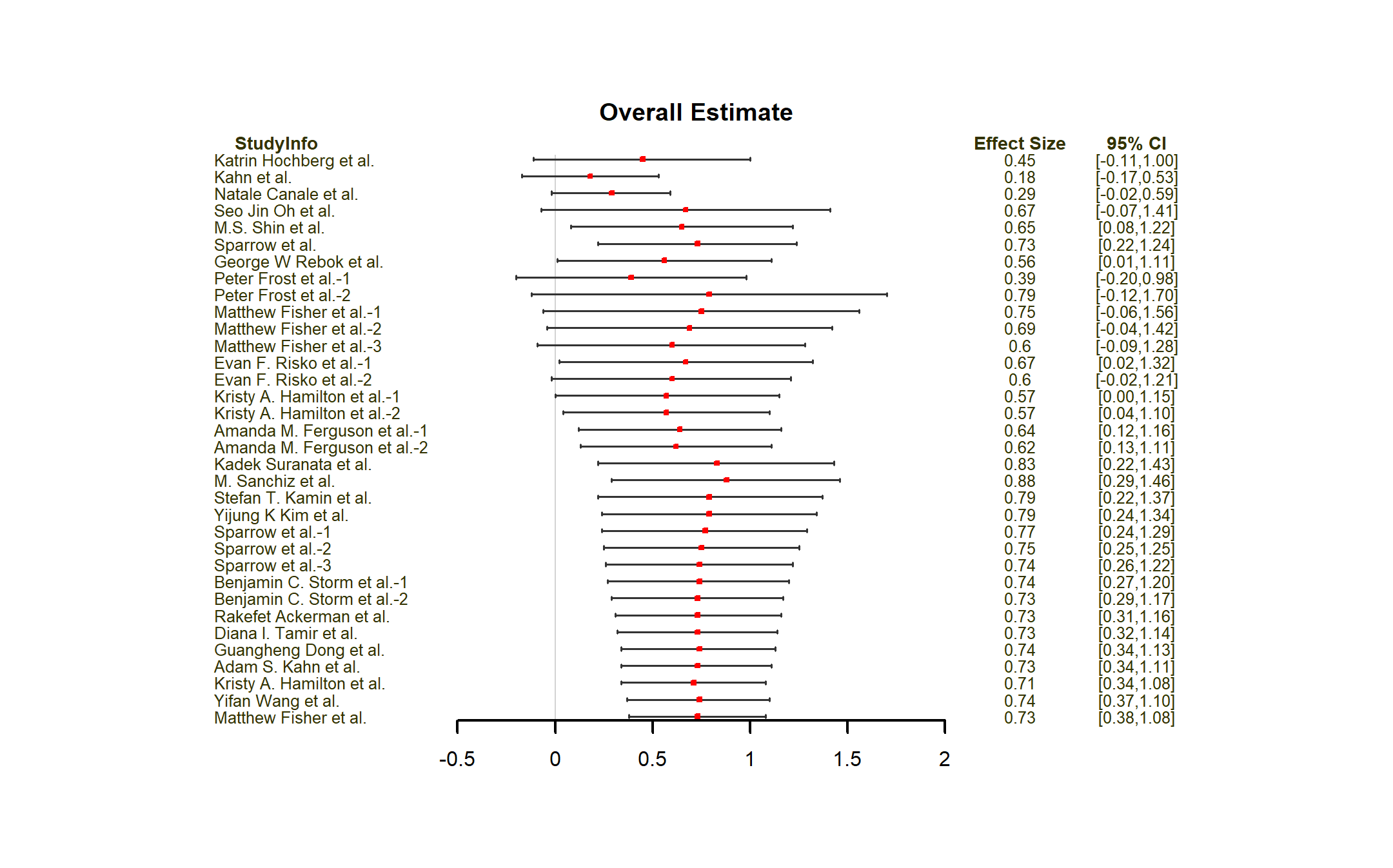


Code for Overall meta analysis:

library(metafor)

library(tidyverse)

names(dat)[16]<-'yi'

names(dat)[17]<-'vi'

names(dat)

res <- rma(yi, vi, data=dat, method="DL")

res

res <- rma(yi, vi, data=dat)

res

funnel(res)

trimfill(res)%>%

plot()

ranktest(res)

################### Formal Analysis #################################

# List of factors

factors <- dat$Type%>%unique()

dat$Type

# Initialize an empty data frame to store results

results <- data.frame()

# Loop over each factor

for (factor in factors) {

# Create subset dataframe

subset_df <- dat%>%filter(Type==factor)

# Perform subgroup analysis

res <- rma(yi, vi, data=subset_df,slab=Author)

funnel(res)

title(factor)

# Extract results

temp_results <- data.frame(

FullName = factors[factor],

Factor = factor,

Estimate = res$b[1],

LowerCI = res$ci.lb,

UpperCI = res$ci.ub

)

# Append to the results dataframe

results <- rbind(results, temp_results)

}

#Figure 1

ggplot()+

geom_point(data=results,

aes(x=factors,y=Estimate),shape=18,size=5)+

geom_errorbar(data=results,

aes(x=factors,y=Estimate,ymin=LowerCI, ymax=UpperCI),

width= .10,

position=position_dodge(.9))+

geom_hline(yintercept = 0,linetype='dashed')+

labs(x = "")+

theme_bw() +

theme(axis.text.x = element_text(angle = 60, hjust = 1)) +

theme(panel.grid.major = element_blank(), panel.grid.minor = element_blank())+

labs(x = "Influencing factors", y = "Effect size (Cohen's d)")

ggsave('subgroup.png',width = 7,height = 5,dpi = 400)

###########################################################################

# meta regression

res1 <- rma(yi, vi, data=dat,

mods = ~`Percentage of Males`)

res2 <- rma(yi, vi, data=dat,

mods = ~Region)

res3 <- rma(yi, vi, data=dat,

mods = ~Age)

res4 <- rma(yi, vi, data=dat,

mods = ~`Analysis Methods`)

res5 <- rma(yi, vi, data=dat,

mods = ~`Experimental Measurement Indicators`)

summary(res5)

Some Running Screenshots:


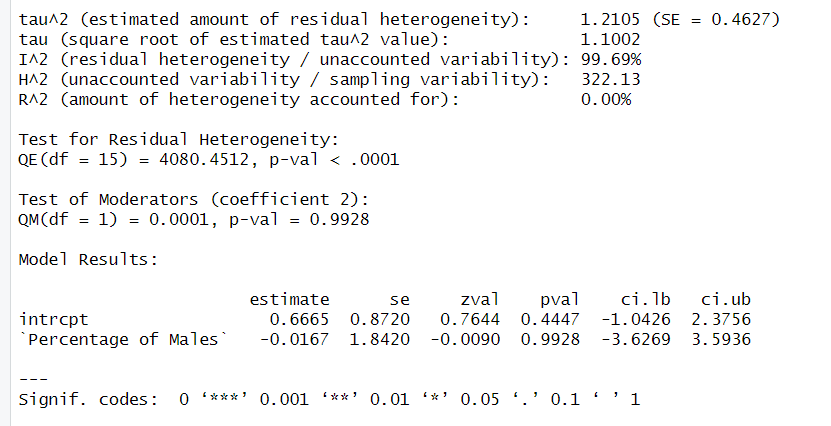


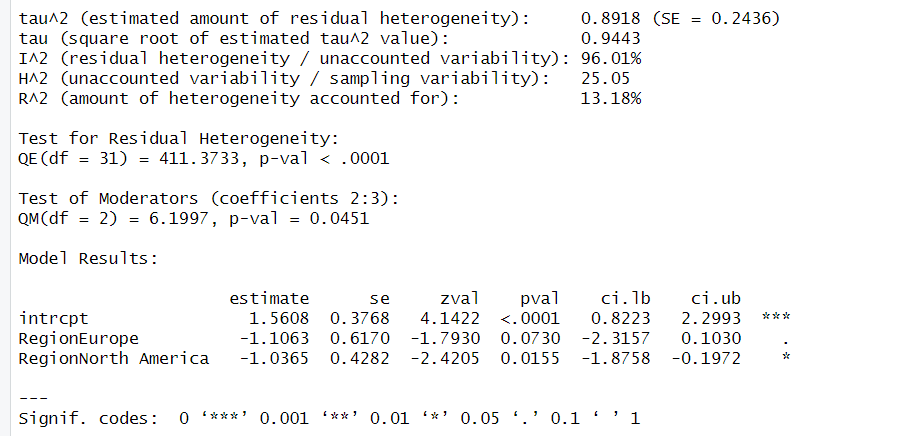


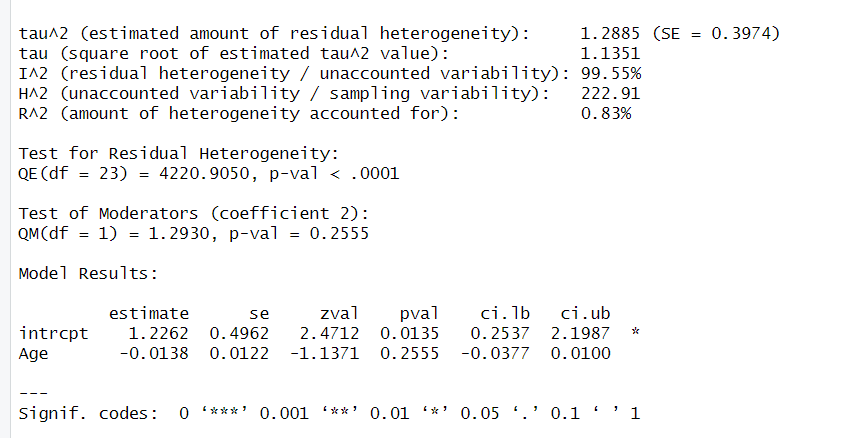

Supplement: Supplementary file 2 [file Data_Sheet_2.DOCX]
